# Study on the blood flow characteristics of venous needle retention with different super-hydrophobic surface structures

**DOI:** 10.1007/s11517-023-02767-5

**Published:** 2023-01-11

**Authors:** Zhun Yu, Lei Liu, Yongzhi Deng, Xiaowen Zhang, Chao Yu

**Affiliations:** 1 Third Affiliated Hospital to Changchun University of Chinese Medicine, Changchun, 130021 Jilin China; 2 Changchun Institute of Optics, Fine Mechanics and Physics, Chinese Academy of Sciences, Changchun, 130033 Jilin China

**Keywords:** Vein retention needle, Blood, Super-hydrophobic surface, Computational fluid dynamics

## Abstract

A venous retention needle, as an implanted device, is very likely to cause thrombosis. In view of the thrombosis phenomenon caused by retention needles, this paper compares the influence of different superhydrophobic surface retentions on blood flow. Compared with other superhydrophobic bulges, the fluid velocity of the four-prism bulge is the highest (0.08 m/s), and the vorticity and shear force of the hemispherical bulge are higher. A large number of vortices can inhibit thrombosis better. The tire vortices generated in the superhydrophobic convex grooves are important vortices to inhibit thrombosis. The enhancement and development of the tire vortex weakens the resistance near the wall of the needle and reduces the probability of platelet aggregation. The superhydrophobic surface structure studied in this paper can not only provide guidance for the design of venous retention needles with better performance but also provide corresponding technical support for the development of human implantation devices.

**Graphical Abstract:**

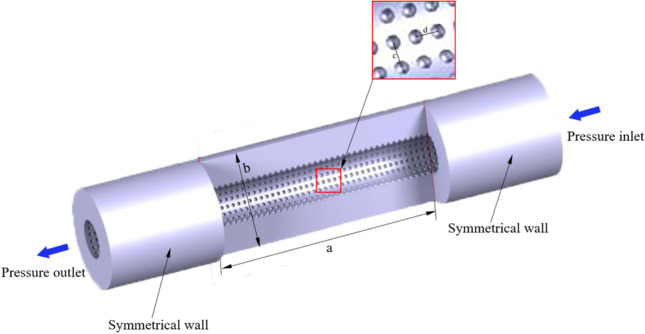

## Introduction

Anticoagulant materials, also known as blood-compatible materials, are biomaterials that do not trigger blood clotting. They are widely used in contact with human blood and tissue, such as blood transfusion catheters, endovascular stents, artificial blood vessels, and others in contact with blood. Under normal circumstances, they will produce varying degrees of coagulation and form thrombi. The formation of a blood clot can block the lumen of the catheter. The clots can cause vascular disease and death in severe cases. As a result, one of the main tasks and central contents of biomaterial research has always been how to improve the anticoagulant performance of materials [1–3]. The super-hydrophobic surface not only has the functions of being waterproof and self-cleaning but also has anti-blood coagulation.

Among all reactions between blood and materials, thrombosis and blood clotting are the most sensitive and complex. Thrombosis is a solid mass formed when blood clots or some components of blood stick to each other (usually in a living heart or vascular cavity) [4, 5]. Thrombosis is related to various blood components such as plasma proteins, clotting factors, and platelets and is a complex chain reaction.

International experts have conducted a large number of studies using in vitro evaluation methods and have developed specific evaluation methods for anticoagulant materials, which usually include platelet adhesion tests, whole blood coagulation tests, hemolysis tests, etc. [6–13]. The researchers believe that the super-hydrophobic surface, with its low apparent free energy and less interaction, shows better anticoagulation. Blood flow characteristics in rough microtubules may also affect its anticoagulant behavior [14–16]. As the flow in normal autologous blood vessels is generally laminar, the rough structure on the super-hydrophobic surface may disturb the flow layer, thus causing the original laminar flow on the wall to be disturbed and inhibiting thrombosis [17]. In vitro static adhesion experiments with platelets after modification of super-phobic and super-hydrophilic surfaces also showed that, compared with the unmodified super-phobic (contact angle = 170°) and super-hydrophilic (contact angle = 10°), the modification brought excellent blood compatibility [4].

Therefore, the formation of thrombosis has an important relationship with the surface structure of the retained needle. Exploring these internal relationships can not only extend the needle's useful life for relieving patient pain, but also provide important guidance for the design of other medical implanted devices.

## Geometric model and boundary conditions

**Fig. 1:**
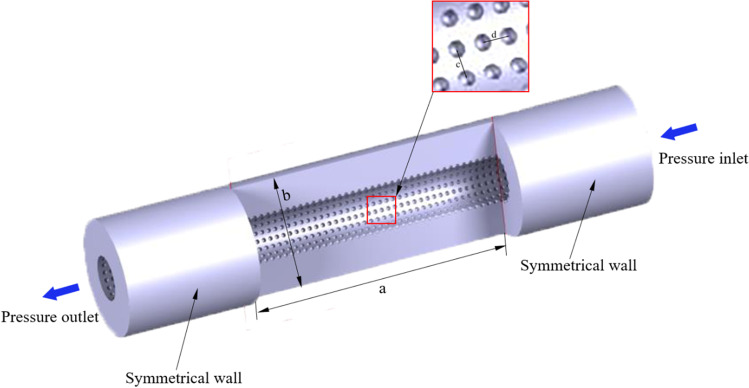
Model of vein retention needle flow field

In this paper, there are three models of super-hydrophobic surfaces proposed. The cube, pyramid, and hemisphere, as the basic shape structures that are easy to process, are selected as the research objects. The needle wall flow fields of three different needles are shown in Fig. 1. Their structural parameters are shown in Table 1. The exit wall of the flow field is symmetric. The inlet and outlet walls are called pressure inlet and pressure outlet, respectively. The CATIA software was used to construct geometry.

**Table 1:** The structural parameter vein retention needle

Structure parameter	Values(mm)	Structure parameter	Values (mm)
Needle diameter	0.8	Hump diameter (cube)	0.05
Flow channel length (a)	8	Hump height (cube)	0.05
Flow channel inner diameter	0.3	Hump diameter (rectangular pyramid)	0.05
Circular array (c)	20°	Hump height (rectangular pyramid)	0.05
Axial array (d)	0.15	Hump diameter (hemisphere)	0.05
Flow channel outer diameter (b)	1	Hump height (hemisphere)	0.05

The blood is a kind of non-Newtonian fluid. The standard k model was used for simulation calculation in order to obtain accurate simulation results. The average time domain of the Navier–Stokes equations to obtain the continuity and momentum equations for incompressible flow is as follows [18–20]:

1 $$\frac{\partial }{\partial t}(\rho k)+\frac{\partial }{\partial {x}_{i}}(\rho k{u}_{i})=\frac{\partial }{\partial {x}_{j}}[(\mu +\frac{{\mu }_{t}}{{\sigma }_{k}})\frac{\partial k}{\partial {x}_{j}}]+{G}_{k}+{G}_{b}-\rho \varepsilon -{Y}_{M}+{S}_{k}$$

2 $$\frac{\partial }{\partial t}(\rho \varepsilon )+\frac{\partial }{\partial {x}_{i}}(\rho \varepsilon {u}_{i})=\frac{\partial }{\partial {x}_{j}}[(\mu +\frac{{\mu }_{t}}{{\sigma }_{\varepsilon }})\frac{\partial \varepsilon }{\partial {x}_{j}}]+{C}_{{1}\varepsilon }\frac{\varepsilon }{k}({G}_{k}+{C}_{3\varepsilon }{G}_{b})-{C}_{2\varepsilon }\rho \frac{{\varepsilon }^{2}}{k}+{S}_{\varepsilon }$$

In the formula, ${\sigma }_{k}=1.0$ and ${\sigma }_{\varepsilon }=1.3$ are the turbulent Prandtl number of *k* and $\varepsilon$ respectively; *S*_*k*_ and *S*_*ε*_ are the source terms; *G*_*k*_ is the turbulent kinetic energy caused by velocity gradient. *G*_*b*_ is the turbulent kinetic energy caused by buoyancy; *Y*_*M*_ is the fluctuation generated by pulsating diffusion in compressed turbulence. The expressions for solving *G*_*k*_, *G*_*b*_ and *Y*_*M*_ are shown in Eqs. (3, 4, and 5):

3 $${G}_{k}=-\overline{{\rho u }_{i}^{^{\prime}}{u}_{j}^{^{\prime}}}\frac{{\partial u}_{j}}{{\partial x}_{i}}$$

4 $${G}_{b}={\beta \mathrm{g}}_{i}\frac{{\mu }_{t}}{{\mathrm{Pr}}_{t}}\frac{\partial T}{{\partial x}_{i}}$$

5 $${Y}_{M}=2\rho \varepsilon {M}_{t}^{2}$$

Among them, $\overline{{\rho u }_{i}^{^{\prime}}{u}_{j}^{^{\prime}}}$ is the rate of change of average momentum; *β* is the coefficient of thermal expansion; *g*_*i*_ is the component of gravitational acceleration; and P_r_ is the Prandtl number. The turbulent Mach number *M*_*t*_ and vortex viscosity coefficient *μ*_*t*_ can be expressed as follows:

6 $${M}_{t}^{2}\text{=}\frac{k}{{a}^{2}}$$

7 $${\mu }_{t}=\rho {C}_{\mu }\frac{{k}^{2}}{\varepsilon }$$

Other parameters in the standard *k*-*ε* model (Formulas (5) and (6)) are as follows: *C*_*μ*_ = 0.09; *C*_*1ε*_ = 1.44, *C*_*2ε*_ = 1.92; *C*_*3ε*_ = tanh∣*v/u*∣; *a*^*2*^ is the kinetic energy of sound propagation in a fluid; *u* and *v* are two mutually perpendicular velocity components.

## Simulation model

**Fig. 2:**
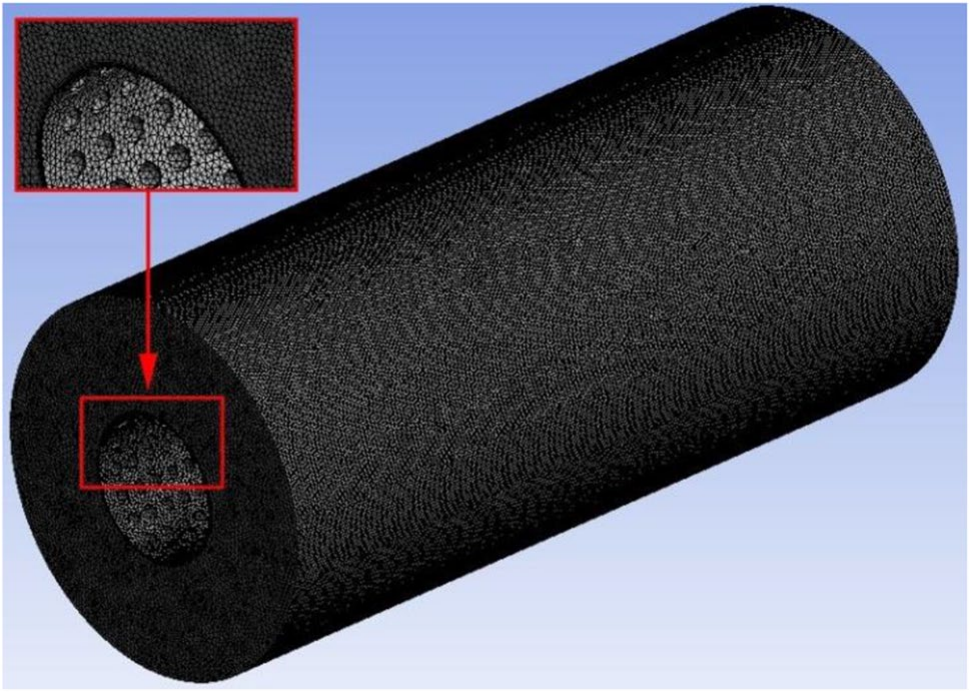
Simulated grid model

**Fig. 3:**
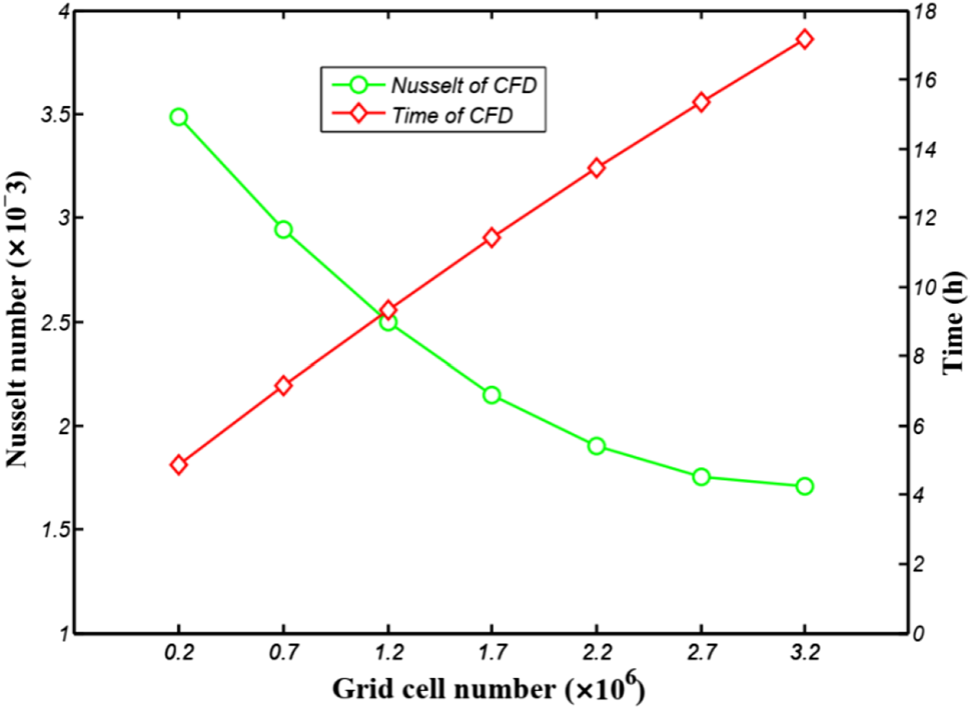
Grid independence verification

Since there are many changes in the internal spatial structure of the flow field, tetrahedral grid elements can be used to divide the solution domain to better fit the grid with the wall surface, as shown in Fig. 2. The software of Workbench and Fluent were used to conduct simulations. In fluid calculation, in order to obtain the optimal grid number of time cost and calculation accuracy, different grid numbers were divided to solve the inlet and outlet pressure difference respectively, and the grid model was selected by comparing the difference between different grid numbers and pressure difference. With the increase of the grid, the computation time gradually increases and tends to flatten out, as shown in Fig. 3. The grid number 2731421 is selected in this research, which can meet the requirements of the venous indwelling needle in this study. The fluid material is set as blood according to the actual usage, and its physical parameters and flow state are shown in Table 2. The flow field inlet is the pressure inlet, and the pressure is 1500 Pa. Flow field outlet is pressure outlet and natural pressure outlet.

**Table 2:** Physical properties and boundary conditions of related materials

Blood	Parameter
Density	1060 kg/m^3^
Dynamic viscosity	0.004 Pa*s
Inlet pressure	1500 Pa

## Performance analysis of super-hydrophobic surface

Since surface-induced blood thrombosis has become a common cause of needle retention complications, improving the blood compatibility of the human needle surface is a major problem in biomaterial science. In this case, good blood compatibility can be achieved simply by controlling the geometry of the surface without changing its chemical composition. We believe that using nature is the best way to solve nature’s problems. Therefore, a super-hydrophobic surface with an ideal size and shape is of great significance [21].

**Fig. 4:**
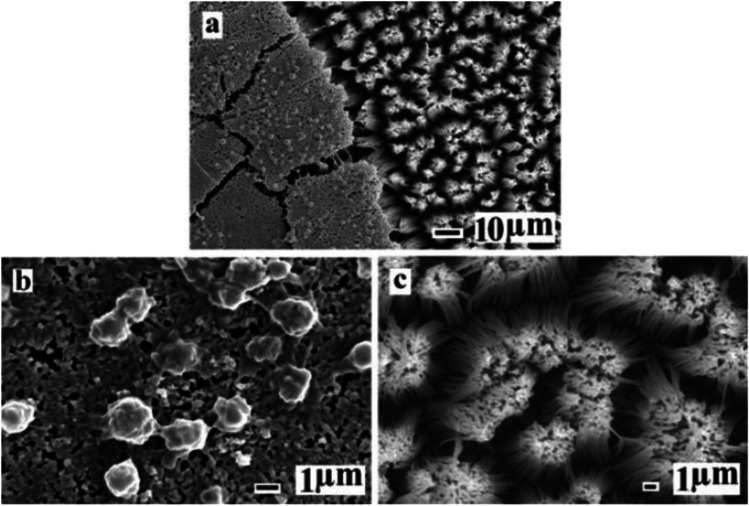
**a** After 90 min of contact with freshly prepared platelet-rich plasma, the two kinds superhydrophobic surface was observed by scanning electron microscopy; **b** enlargement of one structure on the left side; **c** enlarged view of the other on the right side [21]

An interesting phenomenon was also observed in platelet adhesion tests with two different super-hydrophobic surface structures (Fig. 4a). A small number of adherent platelets were observed on the surface of the left super-hydrophobic structure (Fig. 4b). However, it is clear that there is little platelet adhesion on the surface of the right super-hydrophobic structure (Fig. 4c). In conclusion, different super-hydrophobic structures can produce different inhibitory effects on thrombosis.

## Result and discussion

### Influence of super-hydrophobic surface on flow field in needle

**Fig. 5:**
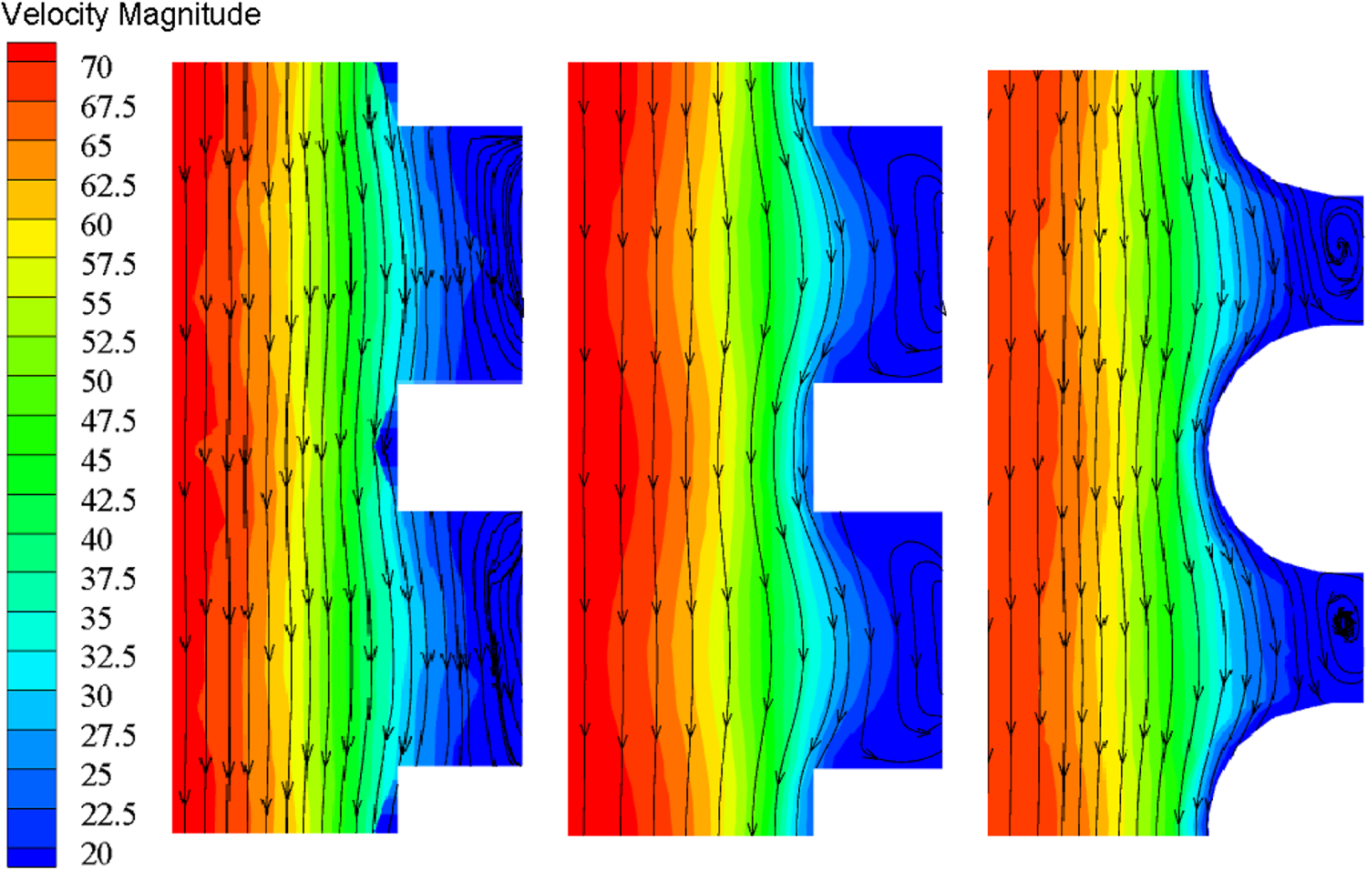
Velocity flow diagram of stranded needle surface (mm/s)

Figure 5 is the velocity flow figure near the wall of the needle with three different structures. By contrast, it can be clearly seen that the fluid velocity with the bionic projection of the cube is higher. Its near-wall velocity is 0.02 m/s, which is 39% and 23% higher than that of the rectangular pyramid and hemisphere, respectively. But for the hemispherical bionic bulge of the needle, the vortex that is formatted in the groove is the most complete. The bionic bulge structure with a rectangular pyramid is inferior to the other two structures in terms of near-wall velocity and vortex formation.

The bionic bulge attenuates the velocity near the needle wall and enhances the velocity far from the center of the needle. So the bionic bulge makes the blood less likely to form thrombosis. When the fluid flow begins to flow through the wall, a part of the blood adheres to the wall due to the viscosity of the fluid, and then the blood flow is blocked and thrombosis is easy to form. As for the bionic retention needle, some special vortex structures will be formed on the surface of the bionic projection when the fluid flows through the needle. They will make the vortex in the flow field constantly upset and re-fit. It is the existence of these bionic bulges that weakens the negative influence of vortex on the blood flow field and realizes the reduction of blood flow resistance. It fundamentally inhibits the formation of thrombosis.

### Influence of super-hydrophobic surface on force in needle

**Fig. 6:**
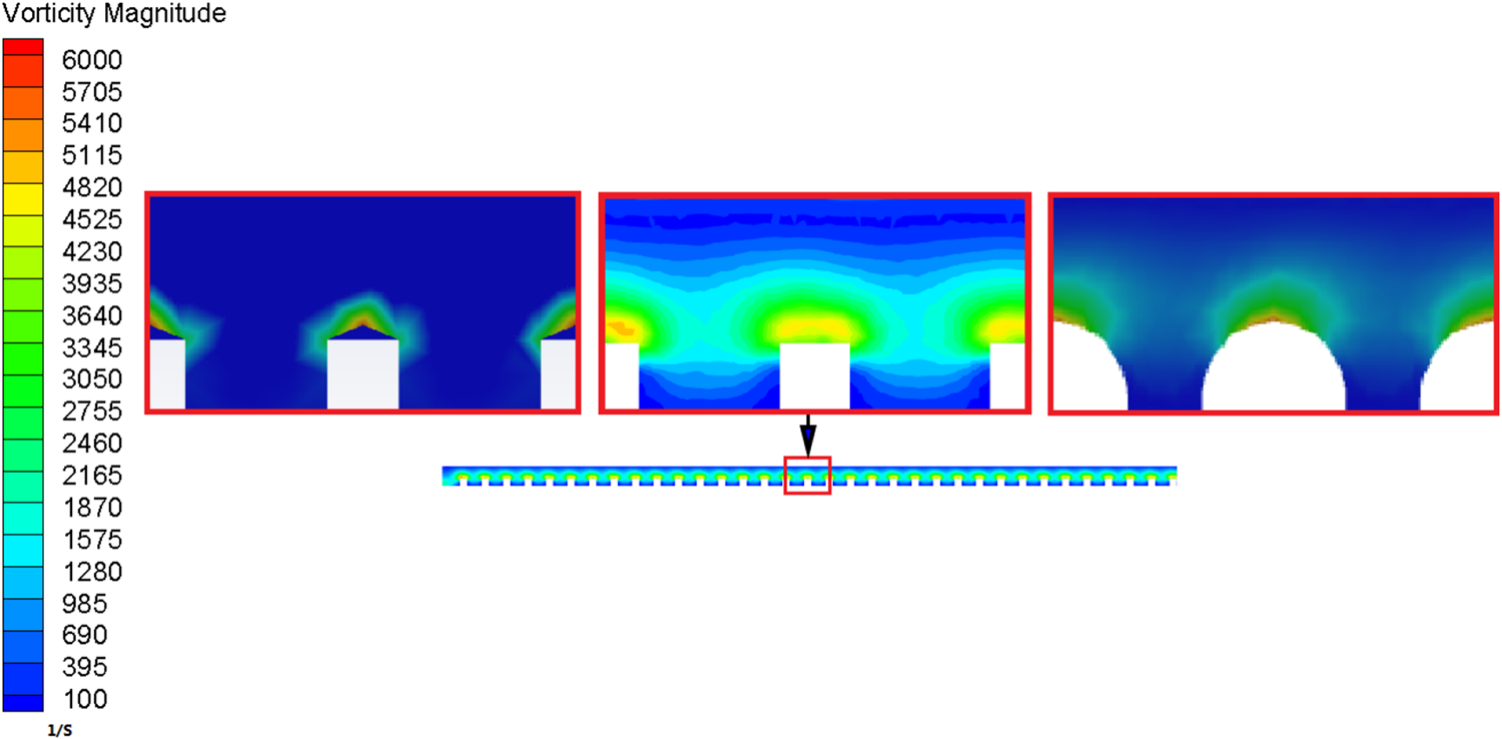
Distribution of vorticity on the wall of needle

Figure 6 depicts the flow field's retention needle vorticity. It shows that the vorticity of blood flow in the rectangular pyramid bionic needle is lower than the other two bulges in the needle. The cube and hemisphere bionic structures are better at forming vortices. And the vortex interferes with most of the velocity and turbulence intensity near the wall. The existence of vortices is beneficial because it shortens the contact time between specific blood particles and the needle wall, which is helpful to inhibit the formation of blood thrombosis.

**Fig. 7:**
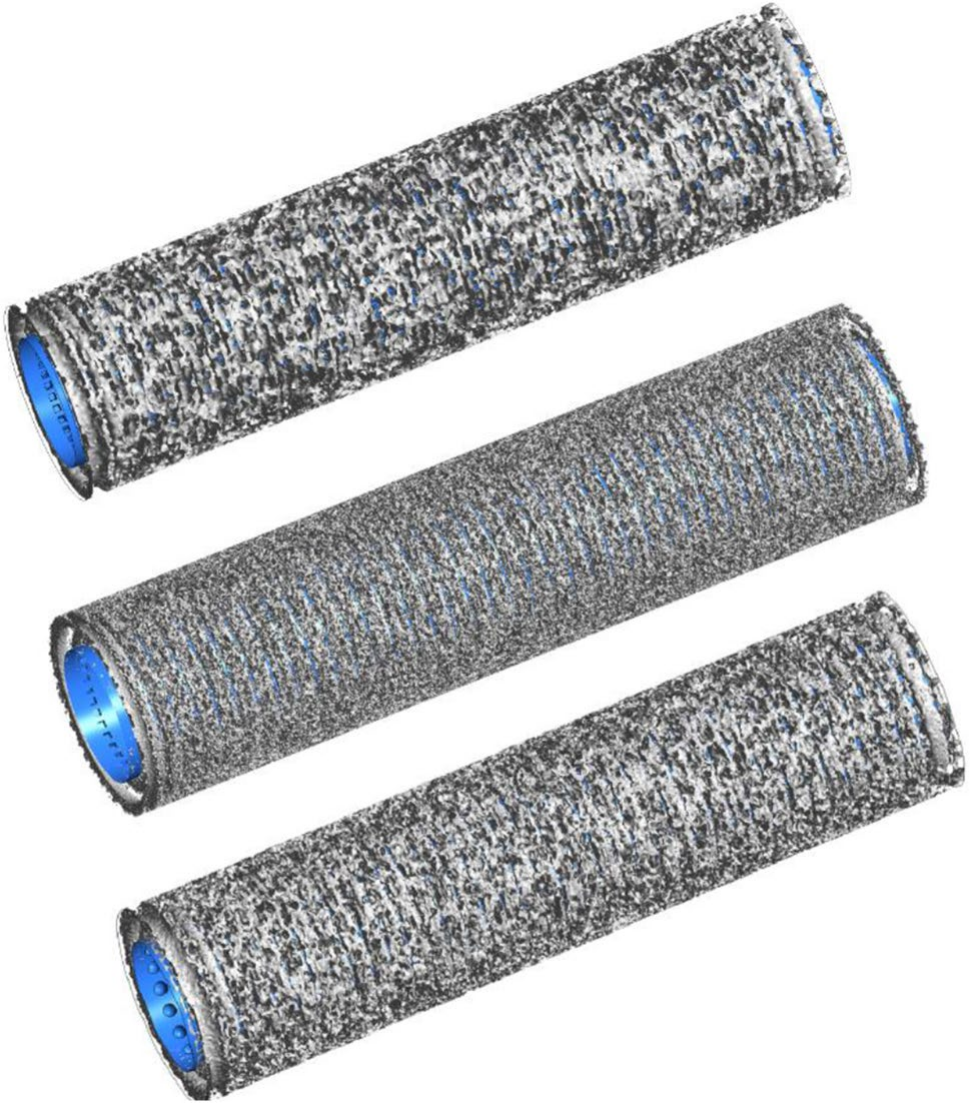
Distribution of vortex core in needle

Figure 7 shows the vortex core distribution figure in the flow area. The vortices near the wall of the hemisphere bulge bionic needle are finer and more uniform, as shown in the figure. The distribution of the vortex core makes the blood-wall contact unstable. This inhibits the coagulation of platelets near the wall and reduces the formation of thrombosis.

### The mechanism of inhibiting thrombosis of super-hydrophobic surface

**Fig. 8:**
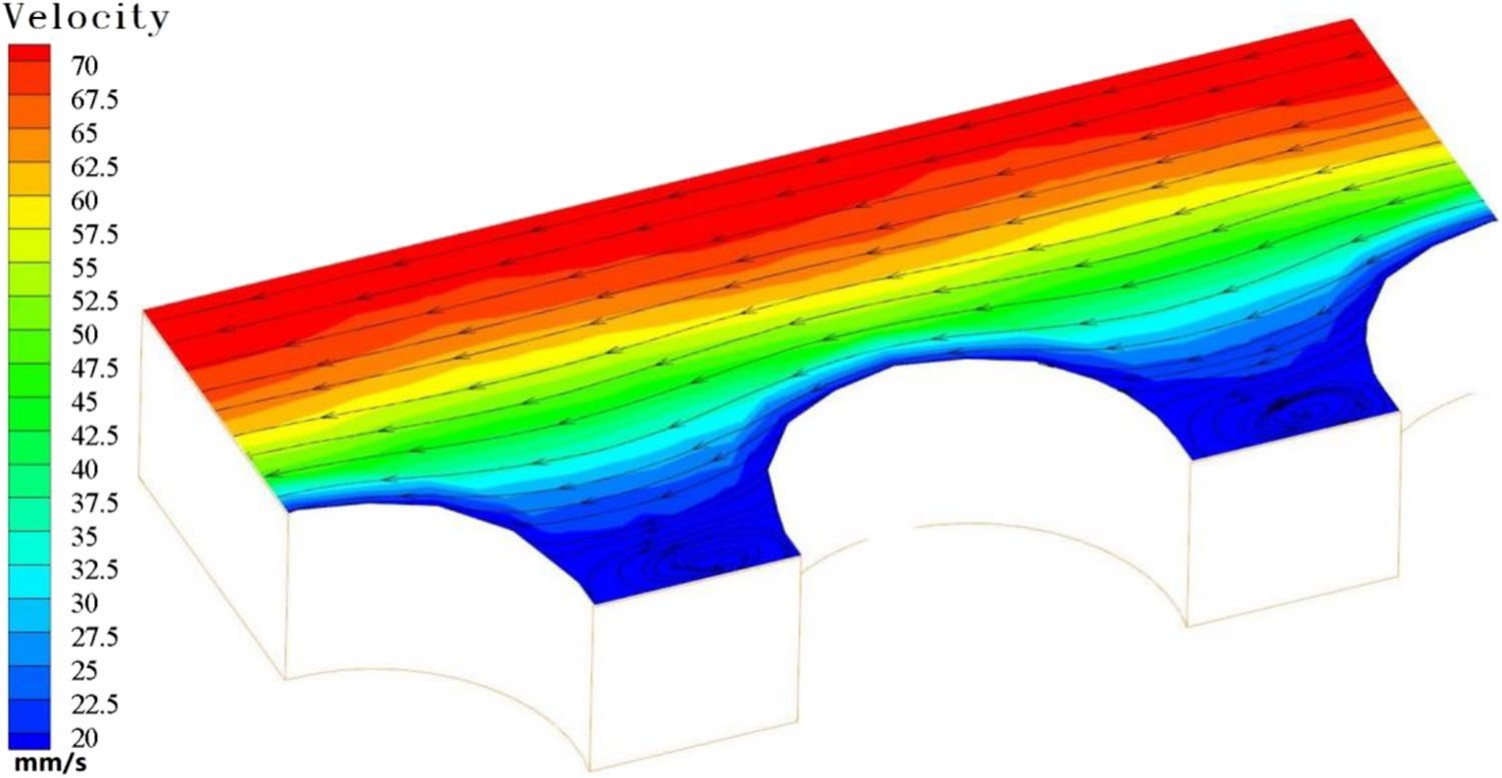
Flow diagram of some areas on the surface near the bulge

As mentioned above, the fluid movement states are not the same on different super-hydrophobic surfaces. This results in various thrombotic inhibition effects. The special flow named “tire vortex” is proposed to analyze and study the conditions near the bulge. The drag reduction effect of the tire vortex in the groove is shown in Fig. 8. The drag reduction mechanism of the vortex is also explained.

The grooves generate vortex flows near the bumps. The vortex is like the tire of a wheel. The vortex that flows through the grooves passes through the spinning tires, thereby reducing losses. It, like the tire, will convert its sliding resistance to rolling resistance, surface contact to point contact, and the vehicle’s running resistance to zero. The flow in the smooth needle boundary layer is completely attached to the wall. However, the tire vortex is near the top of the groove on tires with superhydrophobic surfaces. So the vortex is confined to the bottom of the bionic groove. These tire vortex were critical in separating the vortex's negative influence on the blood flow field. They stabilize the flow field on the needle wall and achieve drag reduction.

In summary, the mechanism of thrombosis inhibition by needle retention on a super-hydrophobic surface is as follows: When blood passes through the surface of the needle tube, flow separation is generated due to its viscosity, and a large number of vortices are generated. When these vortices encounter bionic bulges, tire vortices are produced. With the flow of blood, the tire vortex has been continuously strengthened and developed. That plays a role in inhibiting the original vortex in the blood flow field and its fluid energy. And they also reduce the energy loss in the flow field. The increase in blood velocity reduces the probability of platelet coagulation in the needle wall, which can inhibit the formation of thrombosis. Different surface bulge structures have different effects on the formation and development of vortices. By comparing the three bulge structures, it can be concluded that the vein retention on the super-hydrophobic surface with the spherical bulge has the most obvious inhibitory effect on thrombosis.

## Conclusions

The mechanism of drag reduction and efficiency increase of the bionic retention needle was analyzed and explored by comparing three different bionic protrusion structures, so as to provide a theoretical basis and guiding method for the design of a venous-retained needle to inhibit thrombosis and its complications.

1. Among the three kinds of super-hydrophobic bionic bulges, the fluid velocity near the wall of the cube bionic bulge is the highest, which is 39% and 23% higher than that of the other two bulges, respectively, and its blood flow is smoother. The spherical bionic bulge can effectively increase the number of vortices near the wall of the needle tube. Those vortices can reduce the probability of blood adhesion in the blood flow field and have a better effect on inhibiting thrombosis.
2. In this paper, the “tire vortex” drag reduction mechanism is proposed. The tire vortex effectively separates the negative influence of the vortex in the flow field, stabilizes the flow field on the needle wall, and realizes the inhibition of thrombosis formation.
3. The hydrophobic surface design of the venous retention needle can prolong the deposit time in the wall of the needle and delay the thrombosis of the wall caused by poor blood flow. The design of superhydrophobic surface structures can not only provide guidance for the design of venous retention needles with better performance but also provide corresponding technical support for the development of human implant devices.
